# Global Programme to Eliminate Lymphatic Filariasis: The Processes Underlying Programme Success

**DOI:** 10.1371/journal.pntd.0003328

**Published:** 2014-12-11

**Authors:** Kazuyo Ichimori, Jonathan D. King, Dirk Engels, Aya Yajima, Alexei Mikhailov, Patrick Lammie, Eric A. Ottesen

**Affiliations:** 1 Department of Control of Neglected Tropical Diseases, World Health Organization, Geneva, Switzerland; 2 Division of Parasite Diseases and Malaria, Centers for Disease Control and Prevention, Atlanta, Georgia, United States of America; 3 Neglected Tropical Diseases Support Center, Task Force for Global Health, Decatur, Georgia, United States of America; 4 RTI International, Washington, D.C., United States of America; University of Ghana, Ghana

Lymphatic filariasis (LF) is caused by filarial worms that live in the lymphatic system and commonly lead to lymphoedema, elephantiasis, and hydrocele. LF is recognized as endemic in 73 countries and territories; an estimated 1.39 billion (thousand million) people live in areas where filariasis has been endemic and is now targeted for treatment [1]. Global momentum to eliminate LF has developed over the past 15 years as a result not only of research demonstrating the value of single-dose treatment strategies and point-of-care diagnostic tools, but also of both the generous donations of medicines from the following committed pharmaceutical companies: GlaxoSmithKline (albendazole), Merck (ivermectin), and Eisai (diethylcarbamazine; DEC), and the essential financial support for programme implementation from the donor community [2]. During 2011, more than 50 countries carried out LF elimination programmes, and more than 500 million people received mass treatment [1]. A principal reason for the programme's dramatic expansion and success to date has been the galvanizing of efforts of all key partners around a common policy framework created and coordinated through the World Health Organization's Global Programme to Eliminate Lymphatic Filariasis (GPELF). This report, rather than highlighting the very considerable contributions of each individual partner or even chronicling most of the specific achievements of the GPELF, instead focuses on the details of the underlying processes themselves and their importance in determining programme success.

## Defining the Programme Goals

**Figure 1:**
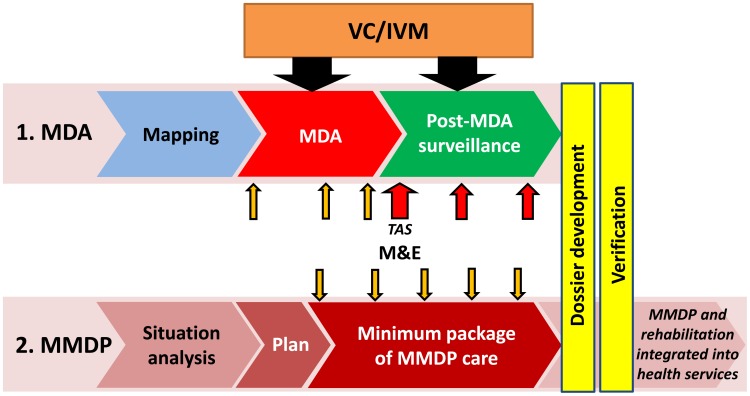
Strategy of the global programme to eliminate lymphatic filariasis. Interrupting transmission through mass drug administration (MDA) and morbidity management and disability prevention (MMDP) in populations with LF [21].

WHO launched the GPELF in 2000 in response to World Health Assembly resolution WHA50.29, which urged Member States to initiate activities to eliminate lymphatic filariasis (LF) as a public health problem, a goal subsequently targeted for 2020. This “global elimination of LF as a public health problem” has been operationally interpreted as the reduction in the prevalence of infection with *Wuchereria bancrofti*, *Brugia malayi*, or *Brugia timori* in all endemic countries to target thresholds below which transmission of the infection cannot be sustained. These thresholds were earlier empirically observed to be less than 1.7% microfilaria (mf) prevalence for Bancroftian filariasis and less than 1.5% mf prevalence for Brugian filariasis [3], though current targets for GPELF are considerably more conservative [4]. In line with its first strategic plan [5], the GPELF has two principal aims: (i) to interrupt LF transmission, and (ii) to manage morbidity and prevent disability [6] (Fig. 1). In 2010, WHO published the GPELF's progress report from its first ten years and a new strategic plan outlining the approach and relevant milestones for its second ten years [2]. The report defines the strategic objective of each of GPELF's two aims as follows:

Interrupting transmission—i.e., providing access to mass drug administration (MDA) for every eligible person in endemic areas where mapping results indicate an infection of greater than or equal to 1%. The main strategy to interrupt transmission for the GPELF is MDA using combinations of two filaricidal medicines (albendazole plus either diethylcarbamazine or ivermectin) delivered once-yearly to entire eligible populations in endemic areas. The MDA aims to reduce microfilaraemia in the blood of infected persons to levels that can no longer sustain transmission of LF by mosquito vectors to new hosts. It should be implemented annually for at least five years, which is generally considered to be the reproductive lifespan of the adult filarial worms in humans [7]–[9].

Morbidity management and disability prevention—i.e., providing access to basic care for LF-related diseases to every affected person in endemic areas. The principal public health impact of LF results from the impairment and disabilities related to lymphoedema, elephantiasis, and hydrocoele. A minimum package of health care aims to treat suffering from acute disease and to prevent disease progression and further disability [10].

With these two components taken together, the GPELF can be seen as a public health programme that provides access to specific health services—MDA and basic care for LF-related disease—for every person in need, thereby improving health for millions of people worldwide. Since LF is concentrated among the poorest segments of society, it is clear that GPELF is also a programme effectively promoting health equity and poverty reduction, in full alignment with the globally accepted Millenium Development Goals [11],[12].

## Establishing a Common Plan: The Policy Framework

Since the publication of GPELF's most recent strategic plan with its clear objectives and milestones towards the attainment of its global elimination goal by 2020 [2], WHO has issued important position statements, technical and policy documents, and guidelines based on newly acquired evidence and updated tools, in order to offer clear guidance to programme managers responsible for LF and other neglected tropical diseases (NTDs). Together, these guidelines provide a common policy framework for the GPELF that each endemic country can rely on to carry out the programmatic steps of its strategic plan en route to achieving elimination at national and regional level (Box 1).

**Box 1. WHO Documents Key to Development of the Underlying Programme Framework and Processes**

**Policy Documents**

- Building Partnerships for Lymphatic Filariasis. Strategic plan [5]
- Progress report 2000–2009 and strategic plan 2010–2020 of the global programme to eliminate lymphatic filariasis: halfway towards eliminating lymphatic filariasis [2]
- Lymphatic filariasis: managing morbidity and preventing disability: an aide-mémoire for national programme managers [21]
- Practical entomology in the global programme to eliminate lymphatic filariasis: a handbook for national elimination programmes [9]

**Guidelines**

- Monitoring and epidemiological assessment of mass drug administration in the global programme to eliminate lymphatic filariasis: a manual for national elimination programmes [23]
- Provisional strategy for interrupting lymphatic filariasis transmission in loiasis-endemic countries: report of a meeting on lymphatic filariasis, malaria and integrated vector management [18]

**Position Statements**

- Transmission assessment surveys in the Global Programme to Eliminate Lymphatic Filariasis [4]
- Position Statement on Managing Morbidity and Preventing Disability in the Global Programme to Eliminate Lymphatic Filariasis [20]
- Position statement on integrated vector management [37]
- Position statement on integrated vector management to control malaria and lymphatic filariasis [38]

### MDA to interrupt transmission

The sequential programmatic steps recommended by WHO [2] for interrupting-transmission are: Mapping the geographical distribution of the disease to determine the need for MDA in each implementation unit (IU: usually a health district), implementing MDA, monitoring for potential resurgence of transmission through surveillance activities for a period of at least five years after MDA has stopped, and verifying the interruption of transmission through a detailed review of historical and epidemiological evidence.

Vector control (VC), also the subject of a GPELF policy document included in this framework [9], is recognized as a powerful tool for supplementing national efforts to interrupt transmission, where its use is both feasible and appropriate [8]. While MDA is the mainstay of control and elimination of LF, success cannot be guaranteed in all situations, and VC can play important complementary roles in LF elimination programmes during both the MDA and the post-MDA surveillance phase [13]. VC can actively reduce the number of vectors available for transmission of the LF parasites while mf are simultaneously being suppressed in infected humans through MDA. In the post-MDA surveillance phase, VC also serves as a strategy to prevent exposure to parasites that might remain in the community and potentially lead to recurrence of transmission [14], [15]. VC also provides the opportunity for direct assessment of LF parasites in vector mosquitoes through PCR techniques, referred to as xenomonitoring or xenosurveillance [16].

In central Africa, a major obstacle to implementing MDA for LF is found in countries coendemic with *Loa loa* because people with high densities of *Loa* microfilariae in the blood are at risk of serious adverse events, including encephalopathy and death, when treated with ivermectin [17]. A 2012 WHO report presents a provisional strategy for interrupting LF transmission in countries endemic for loiasis by implementing intensive VC in combination with twice-yearly treatment using albendazole alone [18]. The WHO Strategic and Technical Advisory Group (STAG) for NTDs (STAG-NTDs) subsequently endorsed this recommendation [19].

### Morbidity management and disability prevention (MMDP) to alleviate suffering

While clearly preventing new infections and likely offering some additional direct benefit, MDA is not designed to treat the lymphedema, elephantiasis, or hydrocoele of affected individuals. Therefore, in order to eliminate LF as a public-health problem, national LF elimination programmes must also implement activities focused on managing morbidity and preventing disability in those already affected by disease. The MMDP component of the GPELF involves a defined sequence of programmatic steps [20],[21]: defining the geographical distribution of LF morbidity and the magnitude of the burden through a situation analysis, developing a detailed plan to provide access to care for all affected persons, providing access to all affected persons through opportunities integrated with other health services.

MMDP activities have lagged behind MDA in terms of both the numbers of countries implementing programmes and the proportion of people needing treatment who have been treated [2]. In recognition of this programmatic need, WHO published a Position Statement on MMDP in 2011 [20]. The MMDP component of the GPELF has the principal aim to provide access to basic recommended care [10], [22] for every person with acute dermatolymphangioadenitis (ADLA), lymphedema, elephantiasis, or hydrocele in all areas where LF is endemic, thus alleviating suffering and promoting improvement in their quality of life [2]. The recommended minimum package of care includes: treating episodes of ADLA/acute attacks among people with lymphoedema or elephantiasis, preventing both the debilitating and painful episodes of ADLA or acute attacks and the progression of lymphoedema or elephantiasis, enhancing access to hydrocele surgery, and providing antifilarial medicines through MDA or individual treatment to destroy any remaining worms and microfilariae.

During the next decade, all countries in which LF is endemic should prioritize building an MMDP component in their national elimination programmes. Guidance for initiating the programme—starting with a situation analysis and planning—and scaling up the activities to achieve full coverage is provided in a recent publication for national programme managers [21].

### Monitoring and evaluation (M&E) to stay on course

During the MDA phase of national programmes, sentinel and spot check site surveys are routinely conducted to monitor the proportion of the targeted population that has received and taken the medicines (programme coverage), as well as the proportion infected (microfilaraemia and/or antigenaemia) in order to determine the effectiveness of MDA delivery [4], [23]–[26]. The major challenge, though, has been to know when the MDAs can be safely stopped. With strong input from the research community [27], a new approach was developed to replace older guidelines that had proved to be unworkable. The revised guidelines, now validated and incorporated into the M&E guidance for GPELF, rely on a new “transmission assessment survey” (TAS) conducted in 6–7 year old children to guide programme manager decision-making for stopping the MDA phase of their LF programmes [23], [27].

A programme area (IU or multiple IUs) is considered eligible for TAS when all of the following criteria are met: (i) at least five rounds of MDA have been implemented, (ii) coverage exceeds 65% of the total population in the IU for each of five rounds of MDA, and (iii) the prevalence of infection in sentinel and spot-check sites is below 1% (assessing microfilaremia) or below 2% (assessing antigenaemia, usually by a rapid card test; ICT). For eligible areas, the design of the survey itself (to identify whether prevalence of infection is below the critical threshold to sustain transmission) is facilitated by a simple, automated algorithm that takes into account a number of variables (including population size, scholarity rates, vector species, and others) [4], [27]. Once an area passes the TAS, it can stop MDA and transition to post-MDA surveillance. Additional rounds of MDA are implemented in areas failing the TAS.

Once MDA has ceased, surveillance is necessary in order to provide evidence that recrudescence has not occurred, and that transmission can be considered as interrupted. Currently, the TAS also serves as the method for post-MDA surveillance. Based on present recommendations, post-MDA TAS should be repeated at least twice at an interval of 2–3 years before beginning the final phase of “verification of the absence of transmission” [4]. Elimination is verified at national level only, and relies on the preparation of a dossier summarizing all LF epidemiologic, programmatic, monitoring, evaluation, and surveillance findings for the country [4]. Intensive research efforts are focused on development and validation of new surveillance tools and strategies (such as the use of antibody assays to reflect exposure to infective larvae and xenomonitoring to confirm absence of parasites in the vectors), both to document the interruption of transmission of LF and to harmonize the process for verifying the elimination of LF with WHO verification processes for other diseases [28].

## Capitalizing on an Integrated Approach

**Figure 2:**
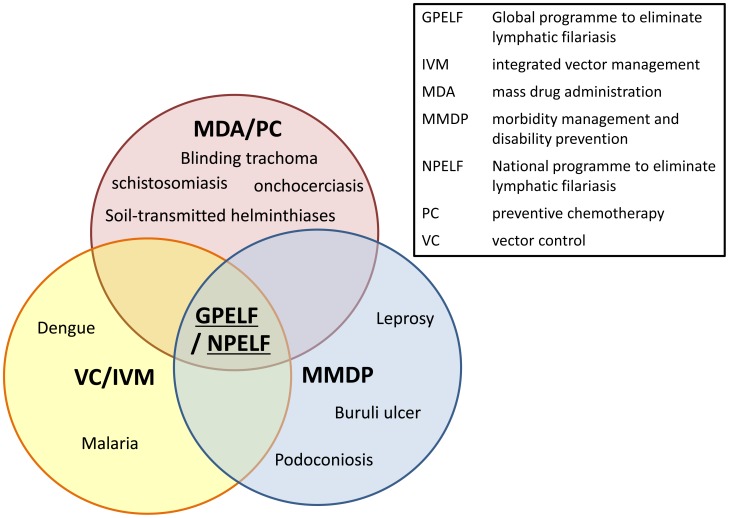
Selected opportunities for integrating lymphatic filariasis activities into programmes for other diseases [21].

The GPELF is now part of integrated efforts to prevent and treat NTDs, in which MDA, VC. and morbidity management are increasingly integrated and delivered as multi-intervention packages at the global, national, and local levels (Fig. 2) [8], [13], [29], [30].

Interrupting LF transmission through MDA and other forms of preventive chemotherapy (PC) is being achieved in collaboration with other NTD programmes [29]–[32]. For example, national LF programmes are now being integrated with those for preventive chemotherapy to control or eliminate onchocerciasis, schistosomiasis, soil-transmitted helminthiasis, and blinding trachoma. This focus on integration is based on the recognition that these diseases are often coendemic and that the programmes targeting them share many common activities—including once- or twice-yearly treatment, advocacy, social mobilization, training of health workers, monitoring treatment coverage, and evaluating outcomes of the interventions—that can be coimplemented. Integrated preventive chemotherapy approaches save costs by optimizing the use of resources across multiple programmes [32], [33].

MMDP activities likely can also benefit from integrated approaches that involve other disease programmes, since the minimum package of care for lymphedema or elephantiasis can address not only LF-related cases but also individuals whose lymphedema or elephantiasis is related to other, non-LF, conditions that affect skin integrity. Possible partners for integration of MMDP activities include programmes focusing on chronic disease, such as those to control leprosy, podoconiosis, diabetes, and Buruli ulcer [10]. Since the basics of the recommended management—skin care, elevation, exercise, and hygiene—are common to all these conditions, regardless of the etiology, integrating management of lymphedema or elephantiasis with that of other chronic diseases requiring long-term care should be both feasible and cost-effective [34]. Even for hydrocoele, a focus on integration could be used to help improve general surgical activities in hospital both qualitatively and quantitatively [22], [35], [36].

It is also essential to recognize that endemic communities will require MMDP beyond MDA, post-MDA surveillance, and even verification of interruption of transmission, since the pathology and damage left behind by the infection will persist for many years. Therefore, GPELF ultimately aims to integrate services for the management of LF morbidity and the prevention of disability fully into national health systems by training health staff to care for these patients, by building on referral mechanisms from community to health worker to specialist and back, and by exploring potential subsidies to help with the cost of treatment [21].

For VC too, Integrated Vector Management (IVM) has now become a recommended strategy across programmes targeting control, elimination or eradication of vector-borne diseases [37]. IVM is defined as a “rational decision-making process for the optimal use of resources for vector control.” The aim is to make a significant contribution to the prevention and control of vector-borne diseases, by marrying conventional single-intervention strategies and promoting multisector approaches to achieve targets cost effectively and promote sustainability. A WHO-published Position Statement on IVM to control malaria and LF [38] recommends implementation of VC as a multidisease approach in areas coendemic for both diseases and where the vectors of malaria and LF are both affected by the same VC interventions (e.g., insecticide-treated mosquito nets, indoor residual spraying, larval control). These recommendations are based on the overlapping geographical distribution of LF and malaria and the fact that *Anopheles* mosquitoes transmit both malaria and LF in many endemic areas [14], [15], [39], [40]. Even where LF is transmitted by other types of mosquitoes, VC measures that target malaria may still reduce transmission of both LF and malaria.

The opportunities presented by such intersectoral and integrated approaches hold the promise of developing even greater synergies among elimination programmes for LF and other programmes targeting vector-borne diseases, and of further extending the benefits of the global LF programme to neglected populations who invariably suffer from multiple overlapping diseases linked to poverty [12], [41].

## Implementing through Partnership

To achieve the Programme's objectives, concerted action will continue to be required. As outlined in the Strategic Plan, key partners must play important roles in helping the national governments and the GPELF overcome its considerable challenges and achieve its global elimination goal [30], specifically the following points:

1. Governments must lead their national programmes to eliminate lymphatic filariasis (NPELF) by developing a national plan of action or including LF in a broader NTD national plan of action, and then by implementing activities of the strategic framework in coordination with in-country, regional and global partners.
2. Nongovernmental organizations (NGO) must support the national programmes by strengthening their capacity and providing technical and logistical expertise to facilitate implementation of national and subnational activities of the governments.
3. Bilateral and multilateral donors will need to provide financial support for programme implementation.
4. Pharmaceutical companies must play an essential role through their continued donation of medicines for MDA and their help in overcoming the logistical challenges associated with drug delivery [42].
5. Academic institutions, WHO Collaborating Centres, and LF Support Centers need to conduct the operational research to provide scientific evidence to guide WHO and national programmes in development of policy to ensure evidence-based strategies and decision making.
6. Donor foundations and government research institutes must provide support to such critical operational research projects.
7. WHO will need to continue to develop and disseminate policies and guidelines, as well as monitor country progress.

Together, all these groups form the Global Alliance to Eliminate Lymphatic Filariasis (GAELF), founded in 2000, which aims to coordinate partners and political, financial, and technical support for the GPELF, and engage in advocacy and fundraising to assist the national programmes [2].

**Figure 3:**
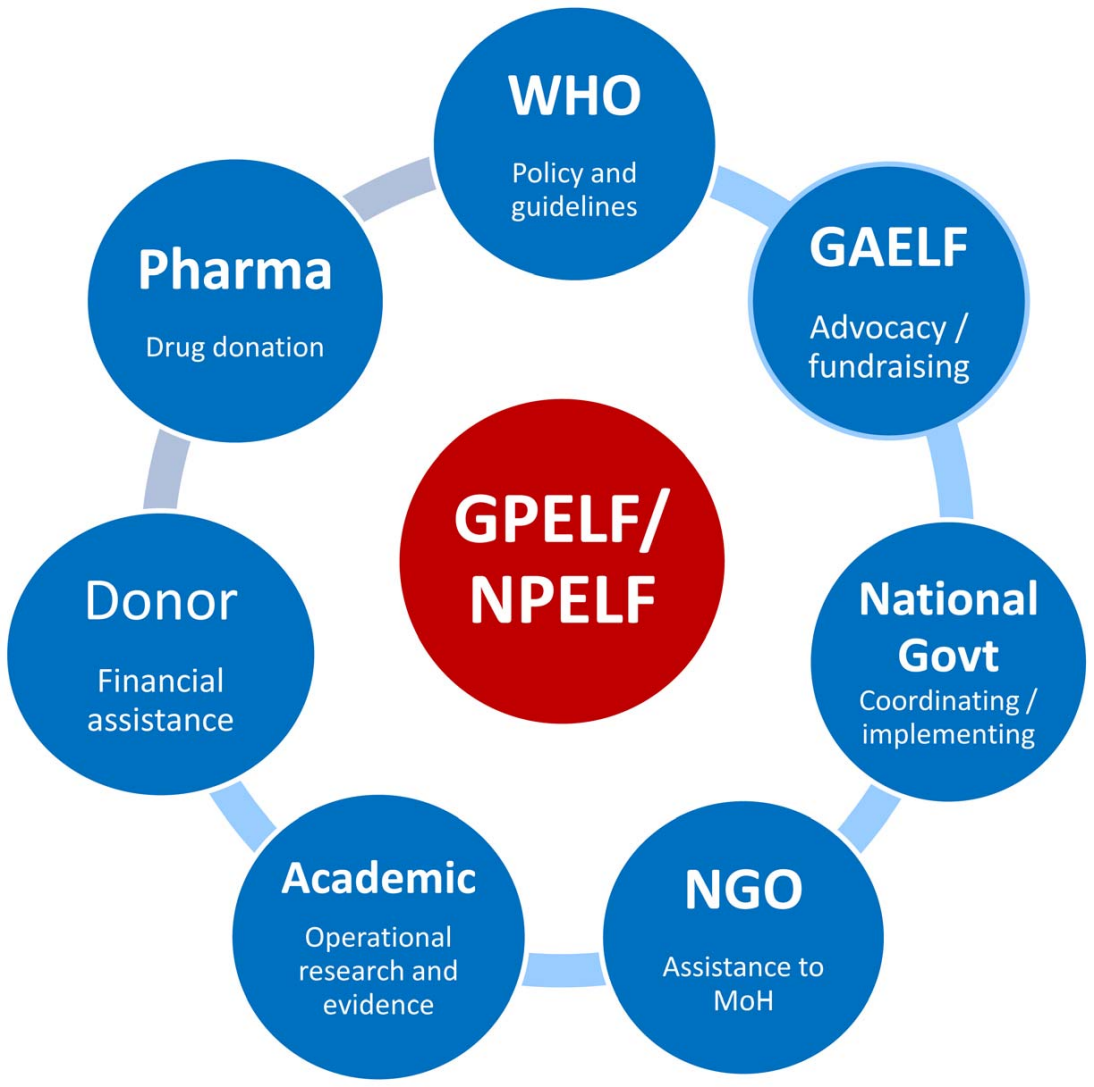
Schematic diagram to demonstrate partnership in GPELF. National govt: National governments; Pharma: pharmaceutical companies.

Fig. 3 demonstrates schematically the extensive partnership supporting the GPELF. Moving forward, all partners will need to coordinate not only among themselves but also with other sectors to ensure the most cost-effective and rational use of resources in support of the elimination of LF.

## Operating under Independent Technical Review

In 1999, WHO established the LF Technical Advisory Group (TAG) to provide expert advice to the GPELF and the LF Programme Review Group (PRG) to monitor the country progress and to address specific technical issues. The PRG was subsequently decentralized to the LF Regional Programme Review Group (RPRG) in 2002 [43]. STAG-NTDs was established in 2007 as the principal advisory group to WHO on overall global policies and strategies for elimination and/or control of NTDs including LF, and, especially, through its working groups, assumed the earlier function of the LF TAG [44]. National programmes now submit annual reports on drug coverage and impact of MDA to WHO, and the results are published in the WHO's PCT Databank that is updated regularly [45]. These independent technical review groups monitor reported data and provide guidance to national programmes. Additionally, these groups review operational research findings to identify the need for new guidelines or modification of current ones.

## Highlighting Success and the Way Forward

WHO recommends that all Member States where lymphatic filariasis is endemic be part of the proposed strategy that aims to reach the elimination goal by 2020. Between the beginning of GPELF in 2000 and 2012, 59 countries have started implementing MDA, and 12 countries have successfully stopped MDA after five or more rounds with high coverage and entered the post-MDA surveillance phase. Of the remaining countries, all 14 will implement and complete LF mapping by the end of 2015.

Through the tremendous efforts of national programmes, GPELF had by 2012 realized the cumulative delivery of more than 3.9 billion doses of medicines to 952 million people covered by MDA worldwide since GPELF was launched in 2000, out of 1.39 billion people in need of treatment [1]. In 2011 alone, over 500 million people were reached; i.e., roughly one in seven persons in the world was receiving MDA targeting lymphatic filariasis. GPELF is recognized as one of the most rapidly expanding global health programmes in public health history and is a remarkable story of partnership where all involved Member States and partners work together aiming at one common goal to achieve remarkable health and economic impact [1], [46], [47].

The GPELF Strategic Plan sets 2014 as the target date for all endemic countries to start MDA, 2016 to achieve full geographic coverage, and 2020 to move to post-MDA surveillance [2]. Within an endemic country, because endemicity is defined and MDA activities implemented at the subnational implementation unit level (usually a health district), at the country level implementation activities begin gradually across districts [30], [32], [48]. Consequently, districts within a country can move along the programme steps at varied rates, and large countries with a greater number of districts require a longer time than small countries, to reach the shared global elimination goal. Taking into account country progress with MDA in each endemic country since 2000 and the size of the population requiring MDA, and assuming that each implementation unit stops MDA after five rounds, the number of individuals needed to be targeted by MDA every year up to the 2020 elimination goal has been projected and updated annually [45]. These optimistic projections of country progress are based on the assumption that each country will be verified for interruption of transmission after five years of post-MDA surveillance.

**Figure 4:**
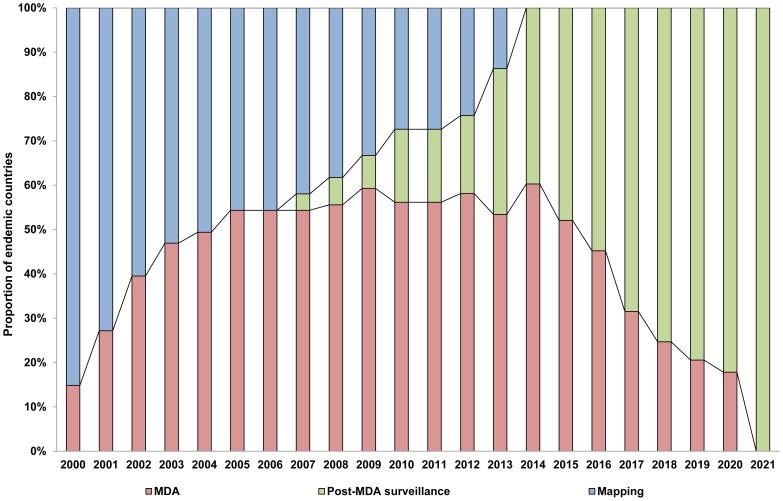
Global view of MDA progress and projection by programme steps, 2000–2021 (updated from [2]).

**Figure 5:**
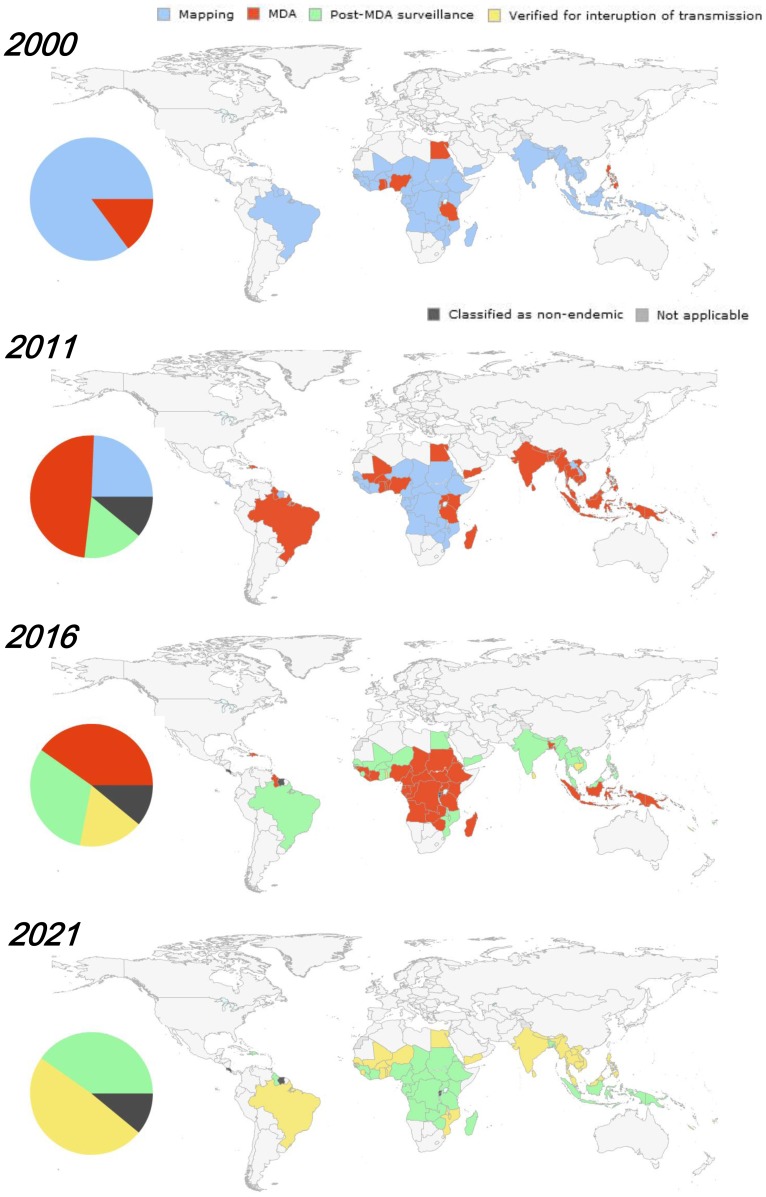
Country-wide MDA progress and projection by programme steps, 2000–2021.

Fig. 4 illustrates the broad view of MDA progress and projection by programme steps from 2000 to 2021, whereas Fig. 5 presents in global maps the country-wide MDA progress and projection by programme steps for the same time period. The first decade of GPELF can be seen to be characterized by rapid scaling up of mapping and MDA; since then, a number of countries have already successfully moved entirely through post-MDA surveillance. In addition, nine out of 81 countries originally identified as endemic at the start of GPELF were reclassified as nonendemic by STAG-NTDs and its working group after review in 2010.

Intensified activities in the next five years will be essential to achieve the global elimination goal by 2020; concerted efforts of the Member States and partners will be required to accelerate (i) completion of mapping and rapid scale-up of MDA in slow-starter and high-burden countries, especially in the African Region, and (ii) progressive implementation of TAS, stopping of MDA and phasing in post-MDA surveillance across the Regions. With this time table, the world should see the final rounds of MDA in 2020—and thus global interruption of transmission of lymphatic filariasis by 2021, even though “verification” of this interruption will still await completion of the post-MDA surveillance in the late-starting countries.
